# No major role for rare plectin variants in arrhythmogenic right ventricular cardiomyopathy

**DOI:** 10.1371/journal.pone.0203078

**Published:** 2018-08-30

**Authors:** Edgar T. Hoorntje, Anna Posafalvi, Petros Syrris, K. Joeri van der Velde, Marieke C. Bolling, Alexandros Protonotarios, Ludolf G. Boven, Nuria Amat-Codina, Judith A. Groeneweg, Arthur A. Wilde, Nara Sobreira, Hugh Calkins, Richard N. W. Hauer, Marcel F. Jonkman, William J. McKenna, Perry M. Elliott, Richard J. Sinke, Maarten P. van den Berg, Stephen P. Chelko, Cynthia A. James, J. Peter van Tintelen, Daniel P. Judge, Jan D. H. Jongbloed

**Affiliations:** 1 Department of Genetics, University Medical Center Groningen, University of Groningen, Groningen, the Netherlands; 2 Durrer Cardiovascular Research Center/Netherlands Heart Institute, Utrecht, the Netherlands; 3 Centre for Heart Muscle Disease, Institute of Cardiovascular Science, University College London, London, United Kingdom; 4 Department of Dermatology, University Medical Center Groningen, University of Groningen, Groningen, the Netherlands; 5 Division of Cardiology, Department of Medicine, Johns Hopkins University School of Medicine, Baltimore, United States of America; 6 Department of Cardiology, University Medical Center Utrecht, Utrecht, the Netherlands; 7 Heart Centre, Department of Clinical and Experimental Cardiology, Cardiovascular Sciences, Amsterdam University Medical Center, University of Amsterdam, Amsterdam, the Netherlands; 8 Princess Al-Jawhara Al-Brahim Centre of Excellence in Research of Hereditary Disorders, Jeddah, Kingdom of Saudi Arabia; 9 Department of Genetics, Johns Hopkins University School of Medicine, Baltimore, United States of America; 10 Department of Cardiology, University Medical Center Groningen, University of Groningen, Groningen, the Netherlands; 11 Department of Clinical Genetics, Cardiovascular Sciences, Amsterdam University Medical Center, University of Amsterdam, Amsterdam, the Netherlands; 12 Medical University of South Carolina, Charleston, United States of America; Indiana University, UNITED STATES

## Abstract

**Aims:**

Likely pathogenic/pathogenic variants in genes encoding desmosomal proteins play an important role in the pathophysiology of arrhythmogenic right ventricular cardiomyopathy (ARVC). However, for a substantial proportion of ARVC patients, the genetic substrate remains unknown. We hypothesized that plectin, a cytolinker protein encoded by the *PLEC* gene, could play a role in ARVC because it has been proposed to link the desmosomal protein desmoplakin to the cytoskeleton and therefore has a potential function in the desmosomal structure.

**Methods:**

We screened *PLEC* in 359 ARVC patients and compared the frequency of rare coding *PLEC* variants (minor allele frequency [MAF] <0.001) between patients and controls. To assess the frequency of rare variants in the control population, we evaluated the rare coding variants (MAF <0.001) found in the European cohort of the Exome Aggregation Database. We further evaluated plectin localization by immunofluorescence in a subset of patients with and without a *PLEC* variant.

**Results:**

Forty ARVC patients carried one or more rare *PLEC* variants (11%, 40/359). However, rare variants also seem to occur frequently in the control population (18%, 4754/26197 individuals). Nor did we find a difference in the prevalence of rare *PLEC* variants in ARVC patients with or without a desmosomal likely pathogenic/pathogenic variant (14% versus 8%, respectively). However, immunofluorescence analysis did show decreased plectin junctional localization in myocardial tissue from 5 ARVC patients with *PLEC* variants.

**Conclusions:**

Although *PLEC* has been hypothesized as a promising candidate gene for ARVC, our current study did not show an enrichment of rare *PLEC* variants in ARVC patients compared to controls and therefore does not support a major role for *PLEC* in this disorder. Although rare *PLEC* variants were associated with abnormal localization in cardiac tissue, the confluence of data does not support a role for plectin abnormalities in ARVC development.

## Introduction

Arrhythmogenic right ventricular cardiomyopathy (ARVC) is a heritable progressive heart condition characterized by fibro-fatty replacement of the ventricular myocardium [1]. ARVC is most commonly transmitted as an autosomal dominant trait [2] and has an estimated prevalence of ~1:2500 [3]. The majority of the likely pathogenic or pathogenic variants variants (>50%) are found in the five genes coding for the major desmosomal proteins: plakophilin-2 (PKP2), desmoplakin (DSP), desmoglein-2 (DSG2), desmocollin-2 (DSC2), and plakoglobin (JUP) [4]. Clinically, ARVC patients suffer from ventricular arrhythmias, syncope, and sudden cardiac death as early as young adulthood [5], with the majority of cases diagnosed before the age of 40 years [6]. Identification of ARVC-associated variants greatly facilitates the identification of family members at risk and provides a better understanding of the underlying pathophysiological mechanisms [1]. However, while more ARVC-associated genes are known [7], they only contribute to a small proportion of cases of this predominantly desmosomal disease, and roughly half of ARVC cases remain gene elusive [8].

We wanted to explore whether the candidate gene *PLEC*, which encodes for plectin, underlies ARVC pathogenesis. Plectin is a large cytolinker protein that belongs to the plakin family of proteins. It is believed to connect the cardiac desmosome to the cytoskeletal intermediate filament desmin via linking DSP within cardiomyocytes [9]. In cardiac tissue, plectin is mainly localized at the intercalated disk and the sarcomeric Z-line, whereas in skin it is located at desmosomes and hemi-desmosomes [10]. This means that plectin potentially has a general and fundamental role in junctional complexes [9]. *PLEC* is a well-known player in the skin disease epidermolysis bullosa simplex with muscular dystrophy (EBS-MD). It is caused by compound heterozygous or homozygous *PLEC* variants, and research on these conditions has led to observations that implicated it in various forms of cardiomyopathy other than ARVC. When fully knocked out in mice, plectin deficiency causes severe skin blistering, generalized skeletal myopathies and ultra-structural abnormalities in the heart [11]. *S*triated-muscle-specific conditional *Plec* knock-out mice showed a decline in endurance performance and, by the age of 16 months, an increase in connective tissue formation in the heart [12]. Sporadic cases of cardiac involvement have also been reported in people with likely pathogenic or pathogenic variants in *PLEC*. These include an EBS-MD patient with ventricular hypertrophy [13]; an EBS-MD patient who, by the age of 30, was discovered to have asymptomatic dilated cardiomyopathy (DCM) that later progressed to right ventricular involvement including (septal) fibrosis [14]; an EBS-MD patient who had a left ventricular non-compaction cardiomyopathy [15]; and a compound heterozygous carrier for two truncating *PLEC* variants who had DCM and episodes of malignant ventricular arrhythmias [16].

Combining PLEC’s potential role in the desmosome with the experimental data and the clinical reports from literature linking EBS-MD and cardiac conditions, led us to hypothesize that plectin may play a role in the pathophysiology of, or increase the susceptibility to, ARVC. We therefore analysed *PLEC* in a large group of ARVC patients, described in previous studies [17–19], and compared this to *PLEC* variants found in the Exome Aggregation Consortium dataset (ExAC) [20]. Cluster analysis was performed to identify possible hotspot regions in *PLEC*. In addition, immunofluorescence analysis was performed on endomyocardial biopsies.

## Results

### ARVC cohort

We identified 47 ‘rare’ (population frequency <0.001) or novel coding variants in Plectin isoform 1 (NM_201380) and three rare variants in two other isoforms (isoform 1a [NM_201384] and 1g [NM_201383]). For an overview of the identified rare or novel coding *PLEC* variants see S1 Table. Plectin has eight isoforms that differ only in the N-terminal sequences encoded by alternatively spliced first exons [21]. The three variants we identified in isoform 1a and 1g were not in included in the subsequent analyses because isoform 1a is not expressed in the heart [22] and isoform 1g does not belong to the muscle-specific set of major isoforms [9].

Of the 359 patients, 40 (11%) carried one or more rare variants: 35 carried one rare variant, four carried two rare variants, and one carried four rare variants. Almost all were missense variants (96%, 45/47), but one was an in frame insertion and one a nonsense variant. There was no significant difference in the percentage of ARVC patients who carried one or more rare *PLEC* variants between those with and without a previously identified ARVC-associated variant (14% versus 8%, respectively [Table 1]).

**Table 1:** Overview of proportion of individuals with one or more *PLEC* variant.

Cohort	ARVC-NL (n = 79)		ARVC-UK (n = 84)		ARVC-US (n = 196)		Total (n = 359)		Fisher’s exact
Rare *PLEC* variant carriers	23% (18/79)		8% (7/84)		7% (15/196)		11% (40/359)		
ARVC LP/P variant	Yes	No	Yes	No	Yes	No	Yes	No	
	n = 42	n = 37	n = 46	n = 38	n = 98	n = 98	n = 186	n = 173	
Rare *PLEC* variant carriers	24% (10/42)	22% (8/37)	13% (6/46)	3% (1/38)	9% (10/98)	5% (5/98)	14% (26/186)	8% (14/173)	P = 0.093

ARVC = Arrhythmogenic right ventricular cardiomyopathy, LP = likely pathogenic, n = number of subjects, P = pathogenic, *PLEC* = plectin

### ExAC Eu

The median coverage of *PLEC* (NM_2013830) in the ExAC Eu cohort was 40x. In total, 4761 rare coding *PLEC* variants were found. S2 Table provides an overview of the identified variants in the ExAC Eu dataset. Seven rare missense variants were found in the homozygous state. Of the 4761 rare variants, 4689 were missense variants (98.5%), 42 were in frame insertions/duplication or deletions (0.9%), and 30 were truncating variants (0.6%). The estimated proportion of individuals with a rare *PLEC* variant (after taking homozygous variants [n = 7] into account) is 18% ([4761–7]/26197).

### Frequency of rare *PLEC* variants in ARVC versus ExAC Eu

The proportion of individuals in the ARVC cohorts with one or more rare *PLEC* variants is 0.11 (Table 1) as compared to 0.18 in ExAC. The case excess of rare *PLEC* variants in the ARVC cohort would therefore be -0.07, showing that there is no enrichment of rare *PLEC* variants in ARVC patients (Table 2). Thirty-three of the 47 rare *PLEC* variants (70%) in the ARVC cohort had a scaled Combined Annotation Dependent Depletion (CADD) score of 20 or more, compared to 3448 of the 4761 *PLEC* variants (72%) in the ExAC Eu dataset [23], indicating no differences in impact of these variants between patients and controls.

**Table 2:** Odds ratio and Chi-Square test results for the proportion of carriers with one or more rare *PLEC* variant in ARVC cases versus ExAC European controls.

ARVC cases			Controls*							
with a rare *PLEC variant*	without a rare *PLEC* variant	Proportion of individuals with a rare *PLEC* variant	with a rare PLEC variant	without a rare PLEC variant	Proportion of individuals with a rare *PLEC* variant	Case Excess	Odds ratio	CI lower	CI upper	Chi-Square
40	319	0.11	4754	21433	0.18	-0.07	0.55	0.39	0.77	0.0002

*ExAC Eu cohort.

ARVC = Arrhythmogenic right ventricular cardiomyopathy, CI = Confidence Interval, *PLEC* = plectin

Ten patients (3%, 10/359) in the ARVC cohort carried one or more unique variant(s) in *PLEC*. Of these 10, six also carried a pathogenic or likely pathogenic variant associated with ARVC. In the ExAC Eu cohort, 500 unique variants were identified, and the estimation of the frequency of unique variants in the European general population would then be approximately 2% (500/26197).

To evaluate the possibility that having a rare *PLEC* variant might negatively modify disease expression, we compared the clinical characteristics of patients who carried an ARVC-associated variant to those of patients who carried an ARVC-associated variant and a rare *PLEC* variant. However, the only difference we found was that patients with an ARVC-associated variant and a rare *PLEC* variant were older at first presentation/evaluation compared to patients carrying only an ARVC-associated variant (S3 Table). There were no differences in other markers of clinical severity, suggesting that the presence of a *PLEC* variant was unlikely to be a negative modifier of disease expression.

### Nonrandom mutation cluster analysis

Of the 47 rare *PLEC* variants, 43 were distinct and four variants were found twice. Mapping these 43 rare variants along the protein sequence and running the non-random mutation cluster analysis with Bonferroni correction revealed no cluster formation at P value <0.1.

### Immunofluorescence analysis

#### Analysis of immunostained myocardial tissue

**Fig 1:**
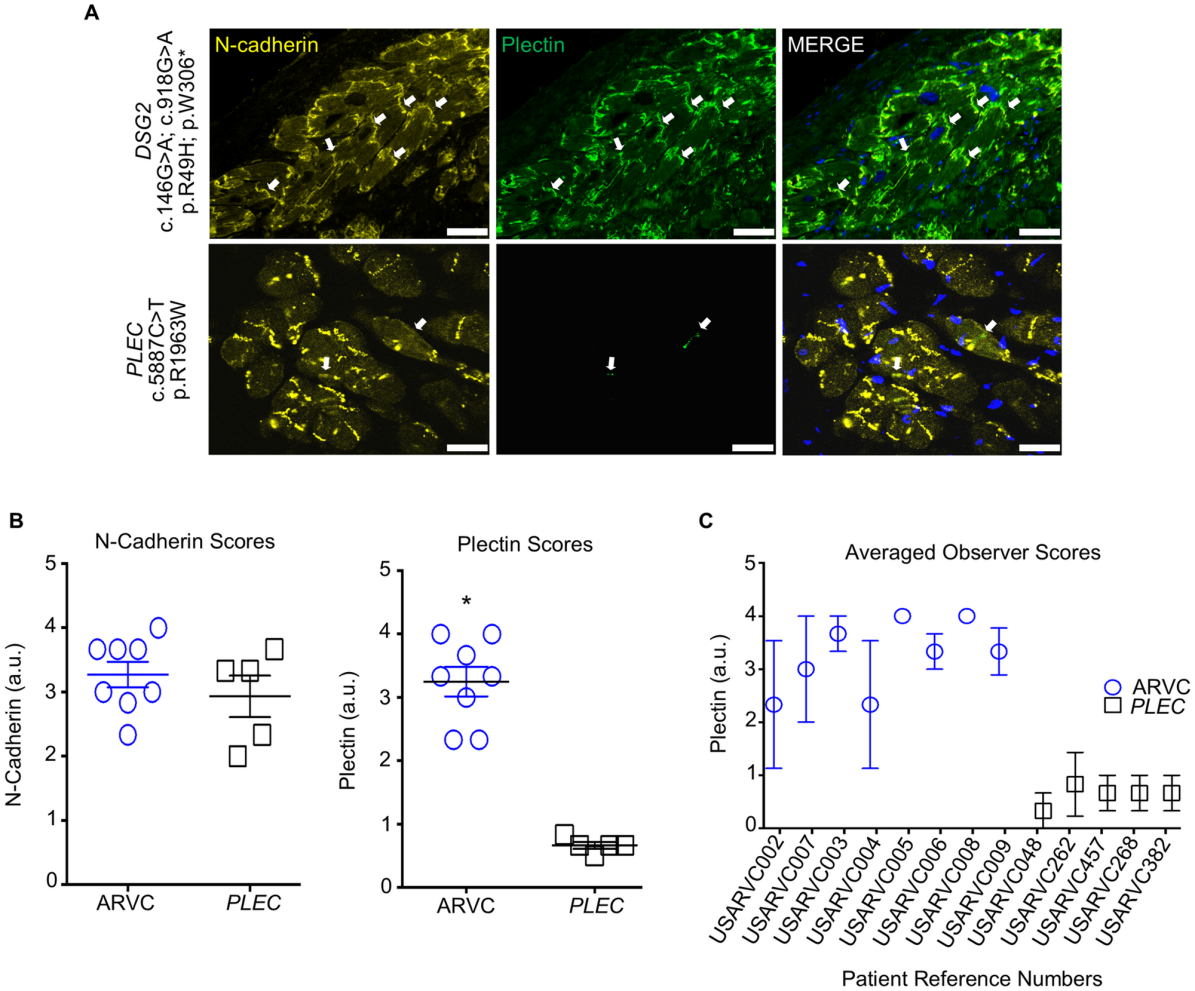
Immunohistochemical analysis of myocardium from TFC+ patients with and without a *PLEC* variant. (A) Representative images of immunostained myocardium from patients from group 1 (n = 8) and group 2 (n = 5) (see also S7 Table). Of note, there is a reduced junctional signal for plectin (white arrows) in myocardium from patients with a *PLEC* variant compared to patients without a *PLEC* variant, even though there is a normal junctional signal for N-cadherin between groups. Scale bar, 25μm. (B) N-cadherin and plectin immunostain scores between groups (n = 3 individual scores were recorded per data point per patient by 3 independent observers). (C) Each patient’s averaged observer scores for plectin. Averaged scores presented as mean±SEM. *P<0.0005 deemed significant for plectin-immunostained myocardium from group 1 versus group 2 using 2-tailed unpaired t-test with equal variances.

Immunostained samples demonstrated predominant junctional N-cadherin localization in myocardium, in both the *PLEC* negative (group 1) and *PLEC* positive (group 2) ARVC patients (*PLEC* negative TFC+ patients, diagnostic score 3.3 ± 0.2 [normal]; *PLEC* positive TFC+ patients, diagnostic score 2.9 ± 0.4 [normal]). *PLEC* negative TFC+ patients displayed robust junctional localization for plectin (diagnostic score 3.3 ± 0.2 [normal]), whereas *PLEC* positive TFC+ patients displayed aberrant junctional plectin localization (diagnostic score 0.7 ± 0.1 [abnormal], Fig 1A and 1B).

Inter-observer reliability analysis (%Agreement) among observers demonstrated an overall inter-observer agreement of 91.7 ± 4.7% and 86.7 ± 9.1% for ARVC TFC+ patients without and with a *PLEC* variant, respectively, for normal N-cadherin junctional localization (diagnostic scores ranging from 2–4). Observers demonstrated an overall inter-observer agreement of 87.5 ± 5.2% for normal plectin junctional localization (diagnostic scores of 2–4) for ARVC patients without an additional rare *PLEC* variant. Whereas, inter-observer agreement was 100.0 ± 0.0% for abnormal junctional plectin localization (diagnostic scores of 0–1) in myocardium for ARVC patients carrying an additional *PLEC* variant (Fig 1C, S4 Table), indicating that the observation of abnormal localization is not due to variation between the observers.

## Discussion

Plectin is a cytolinker protein thought to link the cytoskeleton to the cardiac desmosome. Since the desmosomes play a key role in the pathophysiology of ARVC, we hypothesized that ARVC patients might carry genetic *PLEC* variants that contribute to susceptibility to ARVC. Based on our findings of similar frequency and location of rare *PLEC* variants in both patients and controls and no striking difference in phenotypes in patients with and without *PLEC* variants, we conclude that *PLEC* variants do not play a major role in ARVC pathogenesis. Our immunofluorescence study, however, appeared to show different plectin localization in cases with *PLEC* variants, which may suggest that some variants have an effect in the heart on a molecular level.

### Rare variant frequency in patients and controls

We identified 47 rare or novel heterozygous *PLEC* variants in isoform 1 in TFC+ ARVC patients, and the majority of these were missense variants. Forty (11%) of all patients carried a rare protein-altering variant. However, based on the frequency of rare variants in controls (ExAC Eu), it appears that many individuals in the control population also carry a rare variant (~18%). In the context of these population data, it does not seem likely that *PLEC* plays a major role in ARVC pathophysiology. This is further underscored by the fact that our cluster analysis did not reveal any regions with rare variants that cluster in patients. We also saw no differences in the predicted impact of protein alterations between variants identified in patients or in controls, as comparable percentages of variants with CADD scores >20 were found in both. This is consistent with recent work by Walsh et al showing that some cardiomyopathy-related genes (*MYBPC3*, *MYH6*, and *SCN5A*) show little or no excess burden of variants in DCM patients [24]. This suggests that most variants in these genes are not associated with DCM, although we know that some specific variants are definitely involved [24]. We therefore cannot exclude that some identified *PLEC* variants may have an effect on the development of ARVC or another type of cardiomyopathy. This was, for example, suggested in a patient with skin blistering and DCM, who was compound heterozygous for a truncating and missense variant in *PLEC* [14]. As skin blistering in these patients is generally related to homozygous or compound heterozygous *PLEC* variants, this suggests that in this case the missense variant does play a role and contributes to the phenotype. Moreover, a missense variant in exon 31 of *PLEC* has been described to cause an autosomal dominant form of skin disease, EBS-Ogna [25], which manifests exclusively as skin fragility. This missense variant leads to selective proteolysis of isoform 1a, and thus deficiency in this isoform, resulting in reduced levels and dysfunction of hemi-desmosomes [26]. Similar to what occurs in EBS-Ogna, it could be that some other rare *PLEC* missense variants increase the proteolysis of plectin in the heart and thereby diminish the mechanical coupling. Moreover, heterozygous missense variants were also recently shown to play a role in EBS [27]. Our patients, however, exhibited no striking skin blistering.

### Clinical characteristics

The older age at presentation/evaluation and similar occurrence of life-threatening arrhythmias during presentation/evaluation in the cohort of patients who carry an ARVC-associated variant and a rare *PLEC* variant compared to that of the cohort of patients carrying only an ARVC-associated variant does also not support a major role for *PLEC* as a negative disease modifier in ARVC. In fact, a possible protective effect of carrying a rare *PLEC* variant cannot yet be ruled out.

### Immunofluorescence: Evidence for mechanistic effects

In comparison to our other results, our immunofluorescence analysis showed abnormal localization of plectin in hearts of patients with a *PLEC* variant. As localization of plectin was normal in ARVC patients with or without a desmosomal likely pathogenic/pathogenic variant (*PLEC*-variant negative), abnormal localization of this protein is not a general phenomenon in ARVC patients. Moreover, the fact that the *PKP2* pathogenic variants, c.2146-1G>C and c.148_151delACAG, p.Thr50Serfs*61, were present in both ARVC subgroups (those with and without a rare *PLEC* variant) studied with immunohistochemistry suggests that abnormal plectin localization is also not specifically related to desmosomal variants. In a previous report on EBS patients carrying heterozygous missense *PLEC* variants, immunofluorescence analyses of skin biopsies also showed a decreased signal for plectin [27]. Taken together, these results suggest that the presence of rare *PLEC* variants is accountable for abnormal plectin expression and localization, although this mislocalization may not be sufficient to directly cause ARVC.

In the skin, plectin is a crucial component of the hemidesmosomes (HDs), and HDs connect the epidermis to the extracellular matrix [9]. Plectin insufficiency in the skin results in reduced number of HDs and skin fragility [28]. In the cardiomyocyte, the binding partners of plectin are less well known. *In vivo* studies have shown that plectin is closely associated with desmosomes, while *in vitro* analyses have shown that plectin molecules are biochemically connected to desmoplakin [29]. In addition, the loss of co-localization of plectin and desmin at the Z-discs and intercalated disc, together with desmin and plectin aggregate formation, observed in the heart of the patient with EBS-MD and DCM indicates that plectin plays an important role in the structural organization of the desmin network and in providing mechanical support [14]. We know that plectin contains a number of plakin repeat domains, typical of desmosomal proteins and responsible for binding intermediate filaments, as well as a highly variable N-terminal actin-binding domain [30]. By binding both intermediate filaments and myofilaments, it is possible that plectin attracts the structurally robust desmosomes around the more fragile adherens junctions at the cardiac intercalated disks and thus provides the continuity between myofibrils of neighbouring cardiomyocytes. The abnormal localization of plectin seen in our immunofluorescence analysis could reflect the decrease of its interjunctional linking capacity, which could mean the adherens junctions are less supported by desmosomes and thus more sensitive to stress and damage. One can theorize that this could be a factor that lowers the threshold for developing ARVC. However it is unlikely that these changes on their own are responsible for the development of ARVC, since some of the variants we analysed with immunofluorescence also occurred relatively frequently in the general population (S7 Table), and two occurred even in homozygous state. This, however, does not mean these variants do not have an effect. A heterozygous missense variant that is found relatively often in the European population (MAF = 0.081%) demonstrated a decreased signal for plectin in the skin of a patient with EBS [27]. Additionally, the *PLEC* missense variant (c.1298G>A) described in the EBS-MD patient with DCM has a relatively high MAF (1.9% in ExAC Eu) [14]. This suggests that, in combination with another variant, even a relatively common *PLEC* variant could function as a genetic modifier. In line with this, a recent genome-wide association study identified a missense variant in *PLEC* (MAF 1.2% in Iceland) associated with atrial fibrillation, albeit with a small effect size [31]. However, in order to investigate a putative risk factor/modifier role of *PLEC* variants in ARVC, a much larger sample size is needed. Thus, while the presence of *PLEC* variants in the general population suggest it is not a major player in the pathogenesis of ARVC, it seems that a small sub-set of variants do appear have a mechanistic role that may be additive. Future research on ARVC should keep this in mind when looking at cases with a *PLEC* variant.

## Conclusion

In a large cohort of patients with ARVC, we could not confirm a major role for *PLEC*, a promising candidate gene due to its cytolinking connection to the cardiac desmosome. Some *PLEC* variants, however, are associated with plectin mislocalization, and the association of these changes with ARVC requires further investigation.

## Limitations

We used the ExAC database as a control cohort. However, the ExAC database contains aggregated data, which means it is not possible to evaluate data at an individual level. The frequency of rare variants in *PLEC* that we estimated from ExAC is therefore an approximation based on the assumption that each rare variant is carried by only one individual. This likely an overestimation as we found 88% of our ARVC cohort carried one rare variant while 12% carried more than one rare variant (4 patients carried two variants and 1 patient carried four). However, if we make an estimation assuming that in the ExAC cohort 50% of the individuals carry one rare variant, 25% carry two rare variants and 25% carry three rare variants, the proportion of individuals in ExAC Eu with rare variants would still be 13% (compared to 11% in the ARVC cohort).

The proportion of individuals in the Dutch ARVC cohort with a rare *PLEC* variant is significantly higher compared to the US and UK cohort. This is likely due to the fact that the ExAC Eu cohort for a significant proportion consists of data of individuals from the US and UK. Identified *PLEC* variants in the UK and US ARVC cohorts are therefore more likely to be filtered out, resulting in a lower proportion of these individuals with a rare *PLEC* variant compared to Dutch individuals. Although we also used the Genome of the Netherlands database (n = ~500 individuals) to filter out common variants specific for the Dutch population, the relative small size of this database is likely to lead to an overestimation of rare *PLEC* variants in the Dutch ARVC cohort compared to the US and UK cohort.

As also mentioned above, absence of enrichment of rare variants in patients versus controls does not exclude the possibility that some specific variants may have an effect. To evaluate a possible effect one should assess each variant separately. This would, however, require a high workload of segregation analyses and functional studies in which the chance of finding a significant result would still be low. The immunofluorescence results however are interesting to follow-up. The limitations of these results currently cannot distinguish between abnormal localization due to a general underlying mechanism or abnormal localization due to the specific *PLEC* variant (or combination of variants). To truly obtain more insight we would have to analyse healthy hearts of carriers with rare *PLEC* variants, samples which are not available to us.

## Materials and methods

### Patients

#### Dutch (NL) cohort

Seventy-nine patients were included of whom 75 were diagnosed with definite ARVC according to the revised 2010 Task Force Criteria (TFC+) for ARVC [32]. In three patients, a borderline diagnosis of ARVC was made. A biopsy from another patient showed fibrosis and adipocyte accumulation with atrophy of the myocytes fitting the diagnosis of ARVC (major TFC criterion). The majority of patients were previously screened for variants in desmosomal genes. Some individuals were not screened for all desmosomal genes, either because of the identification of a pathogenic variant in an ARVC-associated gene or because family members with ARVC were shown to be variant-negative for those genes. For a detailed description of the number of patients analysed for the different desmosomal genes see S5 Table. In 42 patients (53%) a likely pathogenic or pathogenic variant associated with ARVC was identified, classified following the guidelines from the American College of Medical Genetics [33]: 29 in *PKP2*, 10 in *PLN*, two in *DSG2*, and one in *SCN5A*.

#### United Kingdom (UK) cohort

The British cohort included 84 TFC+ patients. Forty-six patients (55%) carried a likely pathogenic or pathogenic variant associated with ARVC: 26 in *PKP2*, nine in *DSP*, eight in *DSG2*, one in *DSC2*, one in *LMNA*, and one digenic in *DSC2* and *SCN5A*.

#### United States (US) cohort

In total 196 TFC+ US patients were included. Ninety-eight patients (50%) carried a pathogenic or likely pathogenic desmosomal variant: 76 in *PKP2*, five in *DSP*, seven in *DSG2* (three compound heterozygous), three in *DSC2* (one compound heterozygous), two in *PLN*, two in *SCN5A*, one in *TMEM43*, and two digenic (one in *SCN5A* and *LMNA* and one in *PKP2* and *DSG2*).

#### ExAC European cohort (ExAC Eu)

Genetic variants of *PLEC* (transcript ID ENST00000322810.8, RefSeq NM_201380) were downloaded from the ExAC database (http://exac.broadinstitute.org, version 0.3.1). Since our cohort mainly consisted of patients of European descent (S6 Table), we used the genetic data from individuals of European descent (non-Finnish) in the ExAC cohort (ExAC Eu).

#### Genetic analysis

Genomic DNA was isolated from blood samples using standardized procedures. Written informed consent was obtained from all participants following local medical ethics committee guidelines. The study was approved by the relevant National Research Ethics Service (NRES) committee of the NHS Health Research Authority, the Johns Hopkins School of Medicine Institutional Review Board, and the METc boards of the University Medial Centers of Groningen, Utrecht, and Amsterdam. Our study and all experiments conformed with the principles of the Declaration of Helsinki. In 66 Dutch patients, *PLEC* was analysed by Sanger sequencing. Primers for PCR amplification of the coding regions of the *PLEC* gene were designed to encompass the coding exons as well as adjacent intronic sequences as described previously [27]. Amplifications were conducted following a standard PCR protocol and PCR products were confirmed by direct Sanger sequencing. The remainder of the Dutch patients, the UK and the US patients were all screened with targeted gene panel sequencing (*PLEC* included—list of genes included in panels available on request) or by whole exome sequencing with results then confirmed via Sanger sequencing.

### Data analysis

#### Variant analysis: ARVC cohorts

Chromosomal positions of variants of the *PLEC* gene identified in the three ARVC cohorts (NL, UK and US) were annotated with information from the ExAC database using an in-house developed script and frequency information on these variants were collected. Variant annotation is according to the NM_201380 isoform unless otherwise indicated. All variants found in the ARVC cohorts with a MAF <0.001 in the ExAC Eu dataset were included for further analysis. These included missense, in-frame insertions/deletions, frame-shift, nonsense variants, and variants affecting the consensus RNA splice donor and acceptor sites (first and last two bases of each intron). Additionally *PLEC* variants identified in the Dutch cohort with a MAF <0.001 in the Genome of the Netherlands database (http://www.nlgenome.nl/) were excluded. The NL, UK, and US ARVC cohorts were combined and analysed as one cohort (ARVC cohort, n = 359).

#### Variant analysis: ExAC Eu

Only high-quality (Pass filter) *PLEC* variants found in the ExAC Eu subpopulation were used. All variants (variant types as indicated above) in the designated canonical transcript (NM_201380) with a MAF <0.001 were included for further analysis.

#### Proportion of individuals with a rare *PLEC* variant

The proportion of individuals with rare *PLEC* variants in the ExAC Eu dataset was calculated by dividing the sum of the adjusted allele count by the mean of the total adjusted alleles divided by two, as previously described [24]. The frequency of carriers of rare variants in the ARVC cohort was calculated by dividing the sum of patients with one or more rare *PLEC* variants by the total number of ARVC patients analysed for *PLEC*.

#### Comparison of rare variation between ARVC and ExAC Eu

The proportion of carriers of rare variants in the ARVC cohort was compared with that in ExAC Eu. Case excess was defined by subtracting the proportion of individuals in ExAC Eu with a rare variant from the proportion of individuals carrying a rare *PLEC* variant in the ARVC cohort. We calculated the odds ratio (OR) with 95% confidence intervals.

#### Cluster analysis

To identify putative clustering of rare missense variants in *PLEC*, distinct rare variants were mapped along the protein sequence. Nonrandom mutation cluster, implemented in the iPAC Bioconductor R package, was used to identify clusters of variants in the ARVC cohort [34].

### Immunofluorescence analysis

#### Cohort selection and patient myocardial samples

Endomyocardial biopsies were obtained from 13 US patients. Group 1 (n = 8) consisted of TFC+ ARVC patients with no *PLEC* variant (five with *PKP2* variants, one with compound heterozygous variants in *DSG2*, and two with no pathogenic/likely pathogenic variant identified). Group 2 included TFC+ ARVC patients who either had a variant in *PLEC* alone (n = 3) or a variant in *PLEC* and a likely pathogenic/pathogenic variant in *PKP2* (n = 2). The specific desmosomal variants and *PLEC* variants are documented in S7 Table.

#### Immunostaining myocardial samples

Patient myocardial samples were formalin-fixed, paraffin-embedded, cut at a 5μm thickness and mounted on clear, plus microscope slides. Slides were deparaffinized, rehydrated, underwent antigen retrieval, then blocked at room temperature for one hour as previously described [35]. Slides were incubated overnight at 4°C with mouse anti-N-cadherin (Santa Cruz, sc-59987; 1:500) or rabbit anti-Plectin (Cell Signalling, cs-D6A11; 1:400). The following day, slides were washed and incubated with secondary antibodies (donkey anti-mouse Alexa Fluor-647 [Invitrogen, A31571; 1:500] and goat anti-rabbit Alexa Fluor-488 [Invitrogen, A11070; 1:500]), washed and cover-slipped with mounting media (Fluoroshield with DAPI, Sigma F6057). Immunoreactive signal was visualized using a Leica TCS SPE RGBV confocal microscope (Leica Microsystems) at 40X magnification. Slides were coded and analysed in a blinded fashion by three independent observers.

#### Analysis of immunostained myocardial samples

Slides were imaged, coded, and distributed to three independent, blinded observers and graded as previously described [35,36]. Observers were requested to score samples into one of five diagnostic classes: (0) no myocyte junction plectin staining; (1) rare junction, predominantly cytoplasmic plectin staining; (2) even mix of junction and cytoplasmic plectin staining (odds 50:50); (3) mildly reduced junction, rare cytoplasmic plectin staining; or (4) robust plectin staining, only at cell-cell junctions. Observers were additionally requested to classify patient samples for junctional distribution of N-cadherin using the five diagnostic classes described above. All three observer scores were recorded and averaged for each individual patient (n = 3 different observer scores/patient/immunostain), then averaged by immunohistochemical stain and by group. In addition, observer scores were compared between observers and percent agreement was determined, as described previously [37].

### Statistical analysis

Data are presented as mean ± SEM, and a P value <0.05 was considered significant. Associations between continuous dependent variables were analysed using 2-tailed t-test (binary independent variables) or 2-way ANOVA (2 or more variables). Fisher’s exact test was used for comparing frequencies.

## Supporting information

S1 Table Rare or novel PLEC variants identified in the ARVC cohort (n = 359). (XLSX)

S2 Table Rare PLEC variants identified in the ExAC Eu cohort. (XLSX)

S3 Table PLEC + ARVC-associated variant versus only ARVC-associated variant. (XLSX)

S4 Table Inter-observer analysis of immunostained myocardium and percent agreement between observers. (XLSX)

S5 Table Dutch cohort (n = 79): Genes analysed. (XLSX)

S6 Table ARVC cohort (n = 359): Ethnicities. (XLSX)

S7 Table Information on variant status of patients for whom immunofluorescence staining of heart tissue was performed. (XLSX)
